# Supplementary material for: Hartman effect for spin waves in exchange regime
Source: Sci Rep. 2018 Dec 18;8:17944. doi: 10.1038/s41598-018-35761-1 (PMC6298978; doi:10.1038/s41598-018-35761-1)
Supplement: Supplementary file 1 — Supplementary information [file 41598_2018_35761_MOESM1_ESM.pdf]

Supplementary Information
Hartman effect for spin waves in exchange regime

Jarosław W. Kłos[1,2,*], Yuliya S. Dadoenkova[3,4], Justyna Rychły[1], Nataliya N. Dadoenkova[3,4], Igor L. Lyubchanskii[4,5], and Józef Barnaś[1,6]

[1]Faculty of Physics, Adam Mickiewicz University in Poznań, 61-614 Poznań, Poland
[2]Institute of Physics, University of Greifswald, 17489 Greifswald, Germany
[3]Ulyanovsk State University, 432017 Ulyanovsk, Russia
[4]Donetsk Institute for Physics and Engineering named after O.O. Galkin, NAS of Ukraine, 03680 Kiev, Ukraine
[5]Faculty of Physics, V. N. Karazin Kharkiv National University, 61022 Kharkiv, Ukraine
[6]Institute of Molecular Physics, Polish Academy of Sciences, 60-179 Poznań, Poland
[*]klos@amu.edu.pl

ABSTRACT

Hartman effect for spin waves tunnelling through a barrier in a thin magnetic film is considered theoretically. The barrier is assumed to be created by a locally increased magnetic anisotropy field. The considerations are focused on a nanoscale system operating in the exchange-dominated regime. We derive the formula for group delay $\tau_{\rm gr}$ of a spin wave packet and show that $\tau_{\rm gr}$ saturates with increasing barrier width, which is a signature of the Hartman effect predicted earlier for photonic and electronic systems. In our calculations we consider the general boundary conditions which take into account different strength of exchange coupling between the barrier and its surrounding. As a system suitable for experimental observation of the Hartman effect we propose a CoFeB layer with perpendicular magnetic anisotropy induced by a MgO overlayer.

S1. Boundary conditions for exchange spin waves

The Landau-Lifshitz equation (LLE), describes the dynamics of magnetization $\boldsymbol{M}(\boldsymbol{r},t)$ in an effective magnetic field. Using this continuous model for the description of spin waves (SWs) propagating through the interfaces in magnonic systems, we have to define the relevant boundary conditions. The LLE is a second order differential equation (with respect to the spatial coordinates), which requires two boundary conditions in order to determine the integration constants for a general solution. One of the boundary conditions can be found by integration of LLE in an infinitesimally small surrounding of the interface. The other one has to be postulated using physical principles, which are not inbuilt in the differential equation itself. One of such principles is the conservation of (exchange) energy flux passing through the interface. For a sharp interface between two magnetic materials and in the absence of any interfacial effects (interfacial anisotropy, arbitrary change of the exchange coupling), the boundary conditions (called *natural boundary conditions* (NBC)) for the amplitudes of the dynamical components of the magnetization $\boldsymbol{m}=[m_x,m_y,0]$ (or $m_+=m_x+im_y$ and $m_-=m_x-im_y$) can be formulated in the following form[1–3]:

$$\tilde{M}_{\rm S,l}^{-1} m_\delta(x)\Big|_{x=x_0^-} = \tilde{M}_{\rm S,r}^{-1} m_\delta(x)\Big|_{x=x_0^+}, \tag{1}$$

$$\tilde{M}_{\rm S,l}\lambda_{\rm ex,l}^2 \frac{dm_\delta(x)}{dx}\Big|_{x=x_0^-} = \tilde{M}_{\rm S,r}\lambda_{\rm ex,r}^2 \frac{dm_\delta(x)}{dx}\Big|_{x=x_0^+}, \tag{2}$$

where $x_0=0,L$ are the positions of the interfaces between the matrix and barrier. The indices $\{{\rm l},{\rm r}\}=\alpha$ denote the material parameters on the left hand side (for $x<x_0$) or right hand side ($x>x_0$) of the interfaces, respectively, which can correspond either to the matrix or to the barrier regions . The index $\delta=\{x,y\}$ or $\{+,-\}$ refers to the different components of dynamical magnetization,

By using the NBC, we assume that the magnetic materials are exchange-coupled at the interface without taking into account interfacial effects. Therefore, the following issues need to be answered: how to include the change of an exchange coupling at the interface?; what is actually the *natural* exchange coupling?; when the natural boundary conditions can be applied? These problems were studied in the 70-ties[4] (and discussed more extensively in the 90-ties[5,6]) by introducing the interface exchange energy term and the related contribution to effective field, both for the lattice and continuous models. A new type of boundary conditions was introduced by Hoffmann[7], which are referred to as *Hoffmann boundary conditions* (HBC). The HBC can be

derived on the base of the physical requirement of the continuity of energy flux through the interface[2,3,6], which is also fulfilled by the NBC. These boundary conditions, however, fail in the limit of strong exchange coupling at the interface (including the case of a homogeneous medium, for which the materials on both sides of the interface become identical). The corrected boundary conditions introduced by Barnaś[8] and Mills[9] are referred to as *Barnaś-Mills boundary conditions* (BMBC)[3]. The latter conditions have been used in our paper to calculate the spin wave transmissivity through the barrier of anisotropy field. The general form of HBC and BMBC can also include the impact of different anisotropy fields on both sides of the interfaces. This is especially important in our case where the barrier is formed by anisotropy field.

The BMBC can be written in the following form, which reflects their relation to the NBC and HBC. The first equation reads:

$$D_{\mathrm{l},\beta} m_\delta(x)\big|_{x=x_0^-} = D_{\mathrm{r},\beta} m_\delta(x)\big|_{x=x_0^+}, \tag{3}$$

where the operators $D_{l,\beta} = \left(D^{(1)}_{\mathrm{l},\beta} + D^{(2)}_{\mathrm{l},\beta}\right)$ and $D_{\mathrm{r},\beta} = \left(D^{(1)}_{\mathrm{r},\beta} - D^{(2)}_{\mathrm{r},\beta}\right)$ are expressed by:

$$\begin{aligned} D^{(1)}_{\alpha,N} &= 2\frac{\tilde{M}^2_{\mathrm{S,mb}}\lambda^2_{\mathrm{ex,mb}}}{\tilde{M}_{\mathrm{S},\alpha}}, \\ D^{(2)}_{\alpha,N} &= 0, \end{aligned} \tag{4}$$

$$\begin{aligned} D^{(1)}_{\alpha,H} &= D^{(1)}_{\alpha,N} + t_{\mathrm{l},\alpha}\, t_{\mathrm{mb}}\, \tilde{H}_{\mathrm{a},\alpha}, \\ D^{(2)}_{\alpha,H} &= D^{(2)}_{\alpha,N} + t_{\mathrm{mb}}\, \tilde{M}_{\mathrm{S},\alpha}\lambda^2_{\mathrm{ex},\alpha}\frac{d}{dx}, \end{aligned} \tag{5}$$

$$\begin{aligned} D^{(1)}_{\alpha,BM} &= D^{(1)}_{\alpha,H}, \\ D^{(2)}_{\alpha,BM} &= D^{(2)}_{\alpha,H} - t_{\mathrm{mb}}\frac{\tilde{M}^2_{\mathrm{S,mb}}\lambda^2_{\mathrm{ex,mb}}}{\tilde{M}_{\mathrm{S},\alpha}}\frac{d}{dx}, \end{aligned} \tag{6}$$

where the index $\beta = \{N, H, BM\}$ refers to NBC, HBC, BMBC. Here, the width of the interface between the matrix and the barrier is denoted by t_{mb}, and the introduced interfacial parameter, $\lambda_{\mathrm{ex,mb}}$, is the exchange length in the matrix-barrier interface, whereas $\tilde{M}_{\mathrm{S,mb}}$ is the dimensionless saturation magnetization $M_{\mathrm{S,mb}}$:

$$\tilde{M}_{\mathrm{S,mb}} = \frac{M_{\mathrm{S,mb}}}{H_0}. \tag{7}$$

These parameters are related to the interface exchange stiffness constant: $A_{\mathrm{mb}} = \frac{\mu_0}{2}\lambda^2_{\mathrm{ex,mb}} M^2_{\mathrm{S,mb}}/t_{\mathrm{mb}}$. In turn, the bulk material parameters used here, $\lambda_{\mathrm{ex},\alpha}$ and $\tilde{M}_{\mathrm{S},\alpha} = M_{\mathrm{S},\alpha}/H_0$, can be expressed by the bulk exchange stiffness constant: $A_\alpha = \frac{\mu_0}{2}\lambda^2_{\mathrm{ex},\alpha} M^2_{\mathrm{S},\alpha}$. The parameter $t_{\mathrm{l},\alpha}$ denotes the thickness of the magnetic layer which is different in the matrix ($\alpha = \mathrm{m}$) and in the barrier ($\alpha = \mathrm{b}$). The (effective) anisotropy $K_\alpha = K_{\mathrm{i}}/t_{\mathrm{l},\alpha} - \mu_0 M^2_{\mathrm{S},\alpha}/2$, appearing in the original formulation of the BMBC[8], is related here to the efective anisotropy field by the general formula: $\tilde{H}_{\mathrm{a},\alpha} = 2K_\alpha/(\mu_0 M_{\mathrm{S},\alpha} H_0)$.

The second equation of the boundary conditions is the same for the NBC, HBC and BMBC:

$$D_{\mathrm{l}} m_\delta(x)\big|_{x=x_0^-} = D_{\mathrm{r}} m_\delta(x)\big|_{x=x_0^+}, \tag{8}$$

where the operators D_{l} and D_{r} have the form:

$$D_\alpha = \tilde{M}_{\mathrm{S},\alpha}\lambda^2_{\mathrm{ex},\alpha}\frac{d}{dx}. \tag{9}$$

By inspection of Eqs. (3-9), one can notice that for the BMBC, the components of dynamical magnetization m_x and m_y are continuous[2,8], which is not the case for the HBC. Using the BMBC, we can also correctly determine the values of the interface exchange parameters implicitly existing for the NBC: $A_{\mathrm{mb}}/t_{\mathrm{mb}} = 2A_{\mathrm{m}}A_{\mathrm{b}}/(A_{\mathrm{m}} + A_{\mathrm{b}})$ (see Ref.2) and

$$\lambda_{\mathrm{ex,mb}} = \sqrt{2\frac{M_{\mathrm{S,m}}M_{\mathrm{S,b}}\lambda^2_{\mathrm{ex,m}}\lambda^2_{\mathrm{ex,m}}}{M^2_{\mathrm{S,m}}\lambda^2_{\mathrm{ex,m}} + M^2_{\mathrm{S,b}}\lambda^2_{\mathrm{ex,b}}}}, \tag{10}$$

for $M_{\mathrm{S,mb}} = \sqrt{M_{\mathrm{S,m}}M_{\mathrm{S,b}}}$. Moreover, in the range of weak interface exchange coupling (small A_{mb} or $\lambda_{\mathrm{ex,mb}}$) the BMBC are reduced to the HBC (see Eq. 6).

It is reasonable to assume that thickness of the magnetic layer $t_{\mathrm{l},\lambda}$ (both in barrier and matrix) is smaller than the width of barrier-matrix interface t_{mb}. If additionally $t_{\mathrm{l}\alpha}$ and t_{mb} are both smaller than the exchange length ($\lambda_{\mathrm{ex}} > t_{\mathrm{mb}} > t_{\mathrm{l}\alpha}$), then the term $t_{\mathrm{l},\alpha}t_{\mathrm{mb}}\tilde{H}_{\mathrm{a},\alpha}$ in the boundary conditions (4-6) can be neglected. This simplification allows to derive quite clear and compact analytic formulas for the transmissivity and group delay related to the spin wave transmission through the anisotropy barrier.

S2. The coefficients Δ_s and Δ_c for transmissivity function

The transmissivity function $T(\Omega,L)$ for SWs tunneling through the anisotropy-field barrier can be written in the form:

$$T(\Omega,L)=\frac{e^{-ik_{\mathrm{m}}L}}{\Delta_{c,\beta}\cos(k_{\mathrm{b}}L)+i\Delta_{s,\beta}\sin(k_{\mathrm{b}}L)}. \tag{11}$$

Depending on the boundary conditions, $\beta=\{\mathrm{NBC},\mathrm{HBC},\mathrm{BMBC}\}$, the coefficients Δ_{s} and Δ_{c} can be written in the following forms:

$$\begin{aligned}
\Delta_{\mathrm{c,NBC}} &= 1,\\
\Delta_{\mathrm{s,NBC}} &= -\frac{a^2+b^2}{2ab}, && (12)\\
\Delta_{\mathrm{c,HBC}} &= \Delta_{\mathrm{c,N}}-i\frac{a}{c},\\
\Delta_{\mathrm{s,HBC}} &= \Delta_{\mathrm{s,N}}+\frac{ab}{c^2}+i\frac{b}{c}, && (13)\\
\Delta_{\mathrm{c,BMBC}} &= \Delta_{\mathrm{c,H}}+i\frac{d}{b},\\
\Delta_{\mathrm{s,BMBC}} &= \Delta_{\mathrm{s,H}}+\frac{d^2}{2ab}-\frac{d}{c}-i\frac{d}{a}. && (14)
\end{aligned}$$

The parameters $a=a(\Omega)$ and $b=b(\Omega)$ are the bulk parameters, which depend on $M_{\mathrm{S},\alpha}$ and $\lambda_{\mathrm{ex},\alpha}$; c is expressed only by the interfacial parameters $M_{\mathrm{S,mb}}$, $\lambda_{\mathrm{ex,mb}}$ and t_{mb}; $d=d(\Omega)$ depends on the bulk parameters $M_{\mathrm{S},\alpha}$ and $\lambda_{\mathrm{ex},\alpha}$, and on width of the interface t_{mb}:

$$\begin{aligned}
a(\Omega) &= k_{\mathrm{m}}\tilde{M}^2_{\mathrm{S,m}}\lambda^2_{\mathrm{ex,m}}, && (15)\\
b(\Omega) &= k_{\mathrm{b}}\tilde{M}^2_{\mathrm{S,b}}\lambda^2_{\mathrm{ex,b}}, && (16)\\
c &= \frac{1}{t_{\mathrm{mb}}}\tilde{M}^2_{\mathrm{S,mb}}\lambda^2_{\mathrm{ex,mb}}, && (17)\\
d(\Omega) &= t_{\mathrm{mb}}\frac{k_{\mathrm{b}}a+k_{\mathrm{m}}b}{2}. && (18)
\end{aligned}$$

References

1. Kłos, J. W. & Tkachenko, V. S. Symmetry-related criteria for the occurrence of defect states in magnonic superlattices. *J. Appl. Phys.* **113**, 133907 (2013).
2. Kruglyak, V. V. *et al.* Formation of the band spectrum of spin waves in 1d magnonic crystals with different types of interfacial boundary conditions. *J. Phys. D: Appl. Phys.* **50**, 094003 (2017).
3. Kruglyak, V. V., Gorobets, O. Y., Gorobets, I., Yu & Kuchko, A. N. Magnetization boundary conditions at a ferromagnetic interface of finite thickness. *J. Phys. Condens. Matt.* **26**, 406001 (2014).
4. Hoffmann, F., Stankoff, A. & Pascard, H. Evidence for an exchange coupling at the interface between two ferromagnetic films. *J. Appl. Phys.* **41**, 1022–1023 (1970).
5. Pashaev, K. M. & Mills, D. L. Ferromagnetic-resonance spectrum of exchange-coupled ferromagnetic bilayers. *Phys. Rev. B* **43**, 1187–1189 (1991).
6. Cochran, J. F. & Heinrich, B. Boundary conditions for exchange-coupled magnetic slabs. *Phys. Rev. B* **45**, 13096–13099 (1992).
7. Hoffmann, F. Dynamic pinning induced by nickel layers on permalloy films. *phys. status solidi b* **41**, 807–813 (1970).
8. Barnaś, J. On the Hoffmann boundary conditions at the interface between two ferromagnets. *J. Magn. Magn. Mater.* **102**, 319 – 322 (1991).
9. Mills, D. L. Spin waves in ultrathin exchange-coupled ferromagnetic multilayers: The boundary condition at the interface. *Phys. Rev. B* **45**, 13100–13104 (1992).
